# Newly identified essential amino acids affecting peanut (*Arachis hypogaea* L.) DGAT2 enzyme activity

**DOI:** 10.1016/j.heliyon.2023.e12878

**Published:** 2023-01-17

**Authors:** Zhenying Peng, Ling Zheng, Haiying Tian, Jianguo Wang, Wenwen Liu, Jingjing Meng, Jialei Zhang, Xinguo Li, Shubo Wan

**Affiliations:** a Institute of Crop Germplasm Resources, Shandong Academy of Agricultural Science, Jinan, 250100, China; b College of Life Science, Shandong Normal University, Jinan, 250014, China; c Shandong Academy of Agricultural Science, Jinan, 250100, China

**Keywords:** *Arachis hypogaea* L., DGAT, TAG biosynthesis, Enzyme activity, Fatty acid

## Abstract

Triacylglycerols is the major storage lipid in most crop seeds. As the key enzyme catalyzing the final step of triacylglycerols biosynthesis, the activity of diacylglycerol acyltransferases directly related to oil content. It has been shown that certain amino acids are very important for enzyme activity, one amino acid variation will greatly change the enzyme activity. In this study, we identified three amino acid point mutations that affect the *Arachis hypogaea* diacylglycerol acyltransferase 2 enzyme activity, T107M, K251R and L316P. According to the three amino acid variations, three single-nucleotide-mutant sequences of *Arachis hypogaea diacylglycerol acyltransferase 2a* were constructed and transformed into yeast strain H1246 for function verification. Results showed that T107M and K251R could change the fatty acid content and composition of the transformed yeast strains, whereas L316P led to the loss of enzyme activity. By analyzing the 2D and 3D structures of the three variants, we found that the changes of spatial structure of T107M, K251R and L316P caused the changes of the enzyme activity. Our study could provide a theoretical basis for changing the enzyme activity of DGAT by genetic engineering, and provide a new idea for increasing the oil content of the crops.

## 1 Introduction

Triacylglycerols (TAG) is the major storage lipids in most plants and microalgae for dietary and/or biodiesel. TAG is mainly synthesized by diacylglycerol acyltransferase (DGAT, EC 2.3.1.20), a rate-limiting enzyme of the TAG synthesis pathway [1]. DGATs are commonly divided into four types: DGAT1, DGAT2, wax ester synthase-diacylglycerol acyltransferase (WSD/DGAT), and cytoplasmic DGAT3. Both of DGAT1 and DGAT2 catalyze the final step of TAG synthesis, with no functional redundancy [2–13]. WSD/DGAT predominantly catalyzes the synthesis of wax esters and minor amounts of TAGs, and contributed to drought tolerance in Arabidopsis [14,15]. Cytoplasmic DGAT3 has been reported firstly in peanut and involved in wax synthesis in a variety of plants, and is the first metalloprotein described as a DGAT [[16], [17], [18], [19]].

Given the important function of DGAT1 and DGAT2, researchers have attempted to utilize these genes for crop improvement and biodiesel making. Overexpression of DGAT1 and DGAT2 significantly increases the seed oil content of different plants, especially oil crops [[3], [4], [5], [6], [7], [8], [9], [10],^12^,^13^,[20], [21], [22], [23], [24], [25], [26]]. Seed-specific overexpression of an exogenous *Vernonia galamensis* DGAT1A gene could break the negative linkage of oil and protein contents in soybean seeds, and improve oil yield and nutritional value simultaneously [4]. Characterization of DGAT1 and DGAT2 from the emerging model alga *Chlorella zofingiensis* revealed their functional complementarity and engineering potential [27].

DGAT1 and DGAT2 belong to the membrane-bound O-acyltransferase (MBOAT) family and locate in the endoplasmic reticulum (ER) [2]. It had been known that membrane-bound enzymes are more difficult to study for lacking of knowledge regarding their crystallographic three-dimensional structures. Natural variant amino acid (AA) sites provided us helpful tools to pinpoint the key AA related to the enzyme activity. A nonconservative substitution of lysine by alanine (K232A) of bovine DGAT1 resulted in the decrease of fat content in milk and the change of milk character [28]. Furthermore, A Phe insertion at position 469 of DGAT1 affected the concentrations of seed oil and oleic acid in maize lines [29]. Two DGAT2 gene homologues, CtDGAT2a and CtDGAT2b of an oleaginous yeast *Candida tropicalis* SY005 were identified, and five AA variations were found in of the predicted proteins. 2D and 3D structure analysis showed that many structure differences (such as α-helix and β-sheet) resulted in the enzyme activity of CtDGAT2b was higher than that of CtDGAT2a [22]. We previously identified eight peanut DGAT2 genes (designated *AhDGAT2a-h*) harbouring six natural AA variations in the predicted protein sequences, and two of which displayed increased enzyme activity and/or total cellular fatty acid (FA) content when over-expressed in yeast strain H1246 [30]. But natural AA variations are so few that we cannot get the whole picture of the enzyme active center of DGATs.

Directed evolution is a feasible approach to study the relationship between enzyme structure and function [31]. Directed evolution of DGAT1 was most studied compared with DGAT2. Siloto et al. generated randomly mutagenized libraries of *Brassica napus* DGAT1 (*BnDGAT1*) using error-prone PCR and transformed the mutant sequences into a *Saccharomyces cerevisiae* strain for function verification, and identified some DGAT clones with enhanced ability to synthesize TAG in yeast [32]. Later, Chen et al. investigated the AAs governing BnDGAT1 activity were via directed evolution, and found that numerous AAs were associated with increased BnDGAT1 activity, and 67% of these AAs were conserved among plant DGAT1s [33]. Thereinto two variants I447F or L441P resulted in 33.2 or 70.5% higher TAG content, respectively, compared with native BnDGAT1 [33]. Recently, five newly essential AAs affecting *Chlorella ellipsoidea* DGAT1 function were identified [34]. A directed evolution of a WSD/DGAT from *Thermomonospora curvata* was also reported, the mutant tDGAT has been successfully improved the TAG production with an up to 2.5 times increase in TAG accumulation [35]. These results provide novel insight into the structure and function of DGAT, as well as a mutant gene with high potential for lipid improvement in microalgae and plants.

Up to now, no directional evolution of DGAT2 has been reported. In this study, we obtained a peanut (*Arachis hypogaea* L*.*) *DGAT2* sequence (named as *AhDGAT2i*) using error-prone PCR method. When *AhDGAT2i* was introduced into the TAG-deficient yeast strain H1246, it could not compensate the TAG-deficient phenotype of H1246 and showed no enzyme activity. AhDGAT2i had seven AA differences sites compared with the native AhDGAT2a, and four of which have been identified with no effect on the enzyme activity [30]. Thus, the remaining other three AAs should be closely related to enzyme activity of AhDGAT2i. In order to make sure which one was the deciders, we performed site-specific mutations on these three sites, and transformed the three single-mutant sequences into H1246 for function verification.

## 2 Materials and methods

### 2.1 Sequence alignments of DGAT2 sequences

The AA sequences of AhDGAT2a and AhDGAT2i proteins were compared using DNAMAN software (Lynnon Biosoft, USA). Database searches were conducted using the BLAST program at the National Center for Biotechnology Information (NCBI) database [36] for other plants DGAT2s and aligned using ClustalW software (https://www.genome.jp/tools-bin/clustalw).

### 2.2 Construction of the three mutated variants of AhDGAT2a genes

In order to test the influence of the three AA difference on the biochemical properties of AhDGAT2a enzyme, we created mutated copies of the AhDGAT2a gene. Native AhDGAT2a (Genbank No. JF897614) is regarded as the standard sequence. The three variant sequences are named as T107M, K251R and L316P, respectively.

Overlap-extension PCR was used to mutant the T107M and K251R sites, primers were shown in Table S1 (forward primers AhD2FH/DGAT2T107MR and AhD2FH/DGAT2K251RR, reverse primer DGAT2T107MF/AhD2RH and DGAT2K251RF/AhD2RH). The PCR mixtures (in 30 μL volumes), contained 1 μL KOD Plus Neo polymerase, 1 μL cDNA, 1 μL of each primer (10 μM), 5 μL PCR buffer (10 × buffer), 5 μL dNTPs (2.5 mM each), MgSO_4_ 3 μL, ddH_2_O 13 μL. The reaction was denatured at 94 °C for 5 min, followed by 30 cycles of 30 s at 94 °C, 30 s at 60 °C, 1 min 20 s at 72 °C, then 10 min at 72 °C. The amplified fragments were used as templates to get the full length sequences of T107M and K251R (primers: AhD2FH/AhD2RH were shown in Table S1). L316P was obtained by PCR amplification (primers: AhD2FH/DGAT2L316PRwere shown in Table S1).

### 2.3 Yeast transformation and biochemical activity assay

The ORFs of AhDGAT2a and the three mutant sequences were amplified by PCR, then cut with *Xho* I and *Kpn* I, and cloned into the same sites in multiple cloning sites 2 of the galactose-inducible yeast expression vector pESC-URA (Agilent Technologies, USA). The sequences were labeled with Myc tags in the N-terminus. The plasmids were transformed into the competent cells of *Saccharomyces cerevisiae* strain H1246 and selected on SD-URA media [2]. The colonies from each strain were spotted on galactose solid media lacking uracil, and containing either no FAs, or 1–3 mM oleic or linoleic acid. These cultures were grown at 30 °C under dark and the growth status photographed by a Nikon camera D7000 at 24 h, 48 h, and 72 h. Strains bearing empty pESC-URA plasmid or plasmid which contains *AhDGAT2a* and *Vernicia fordii DGAT2* (*VfDGAT2*, GenBank: DQ356682.1) [2] were used as negative and positive controls, respectively. Simultaneously, these cultures were grown at 30 °C with shaking and the growth rates monitored by OD_600_ readings on a spectrophotometer at 24 h, 48 h, and 72 h.

### 2.4 Reverse transcription PCR (RT-PCR) of the transformed yeast strains

The total RNA of each transformed yeast strains was extracted using PureLink™ RNA Mini Kit (production ID: 12183018A, ThermoFisher). A yeast actin gene (Genbank No. 850504, primers shown in Table S1) was used as the reference gene for RT-PCR. The template dosage was adjusted to equal amounts. The PCR reaction system was prepared as follows: T5 Super PCR Mix 10 μL (Tsingke, Beijing), AhD2FH primer (10 μM) 0.4 μL, AhD2RH primer (10 μM) 0.4 μL, cDNA (100 ng/μL) 1 μL, and ddH_2_O 8.2 μL. The amplification program was as follows: an initial denaturation step that consisted of 3 min at 98 °C, followed by 28 cycles of 5 s at 98 °C, 15 s at 58 °C, 10 s at 72 °C, and extension at 72 °C for 2 min. The PCR products were separated on 1.5% agarose gel and photographed using an AlphaImager EP imaging system (NatureGene Corporation).

### 2.5 Protein extraction and western blot analysis

The transformed yeast strains were cultured on SGal-URA medium [2]. When OD_600_ was approximately 1.0, the total protein was extracted using Yeast Total Protein Extraction Kit (production ID: C500013, Sangon Biotech, Shanghai, China). Protein concentration was detected using BCA Protein Assay Kit (production ID: P0011, Beyotime Biotechnology, Shanghai, China). Subsequently, myc tag antibody (production ID: 66003-1-Ig), and yeast actin antibody (production ID: 60008-1-Ig) were used for Western blot analysis following the user’s manual (Proteintech, USA).

### 2.6 Nile Red staining

Aliquots of stationary phase cells (400 μL) were pelleted and washed twice in PBS, gently dispersed in 20 μL of PBS, then mixed with 5 μL Nile Red (1 μg/μL) [37,38]. The stained cells were incubated in the dark for 10 min at 30 °C, then washed twice in PBS and diluted into 100 μL of PBS. The stained cells were observed and photographed with a fluorescence microscope (Olympus IX71-A12FL/PH, Japan) containing a digital camera. We used Image-Pro plus software (Media Cybernetics, Rockville, MD, USA) to analyze the fluorescence intensity of transgenic yeasts.

### 2.7 Crude lipid extraction and thin-layer chromatography (TLC) analysis

After the transformed yeast strains were grown on SGal-URA medium for 3 days, the cells were placed in 80 °C water for 10 min, collected and washed twice with sterile water, freeze-dried, and weighed. Then, 0.3 g of dried sample was placed in liquid nitrogen and ground into powder. Subsequently, 5 mL of chloroform:methanol (v:v = 2:1) was added, and the sample was kept at room temperature for 3 h. After centrifuging at 3000 rpm for 3 min to collect the supernatant, 1 mL of chloroform:methanol (v:v = 2:1) was added and mixed. After centrifuging at 3000 rpm for 3 min to collect the supernatant, 2 mL 0.9% NaCl was added and mixed. Then, the sample was centrifuged at 3000 rpm for 3 min to collect the lower organic phase. The organic phase was dried by importing nitrogen. Thereafter, 40 mg of extracted lipid dissolved in 1 mL of chloroform was obtained, and 5 μL was measured on a silica gel plate for TLC analysis [30]. The developing agent used was hexane:ether:glacial acetic acid (v:v:v = 70:30:1). Color was developed via iodine vaporization. Pictures were captured with a Nikon camera D7000.

### 2.8 Fatty acid analysis via gas chromatography

Each of the transformed yeast strains were grown in media containing galactose, pelleted by centrifugation and cryodesiccated, accurately weighed, and ground into a powder in a test tube. Three technical replicates were conducted for each sample. The methyl ester of heptadecanoic acid (Nu-Chek, USA) was used as a reference standard. Samples and standard were soaked in 2 mL of 2% sulfuric acid in dry methanol for 16 h at room temperature, followed by 80 min of heating at 90 °C to convert the FAs into FA methyl esters. After addition of 2 mL of distilled water and 3 mL of hexane, the FAMEs were extracted for analysis by gas chromatography (GC). The other information referred to Zheng (2017) et al. [39].

### 2.9 Two- and three-dimensional structure prediction

Two-dimensional (2D) structure prediction was used of Phyre2 software (http://www.sbg.bio.ic.ac.uk/phyre2/html/page.cgi?id=index) with default parameters [40]. The three-dimensional (3D) structure of DGAT2 was predicted using Swiss-Model software online (https://swissmodel.expasy.org/) and analyzed using Swiss-Pdb Viewer.

## 3 Results

### 3.1 Enzyme activity test of AhDGAT2a and AhDGAT2i

**Fig. 1:**
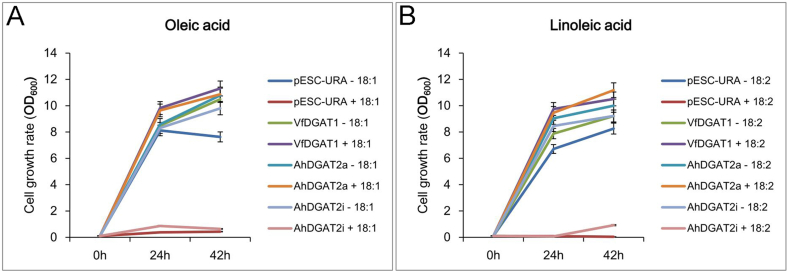
Lipotoxicity rescue assays of AhDGAT2s. Cells of the H1246 were transformed with plasmids bearing AhDGAT2a or AhDGAT2i, then cultured in solid media containing oleic (A) or linoleic acid (B) at 30 °C. VfDGAT2- and pESC-URA- plasmid-transformants were used as positive and negative control respectively. OD_600_ values were detected at different time.

By error-prone PCR we obtained a sequence named as *AhDGAT2i*, and transformed it into a TAG-deficient yeast strain H1246 and selected on media containing 1–3 mM oleic (Fig. 1A) or linoleic acid (Fig. 1B), the transformed strain showed very low growth rate as well as the empty pESC-URA plasmid transgenic strain, indicating that AhDGAT2i had very low or no enzyme activity compared with AhDGAT2a.

**Fig. 2:**
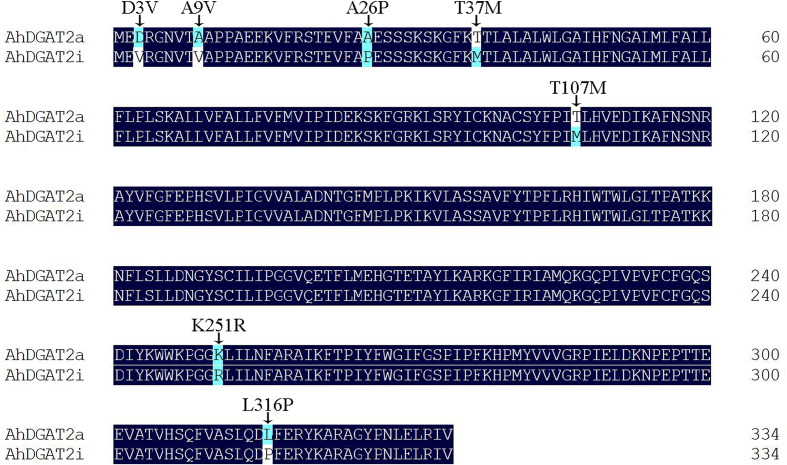
AA sequence comparison of AhDGAT2a and AhDGAT2i. D3V, A9V, A26P, T37M, T107M, K251R and L316P indicate the seven variant AAs, where the first letter represents the consensus residue, followed by the numerical location of the residue, and the letter representing the alternate residue present in the protein variant.

AA sequence alignments inspection of AhDGAT2a (GenBank: JF897614) and AhDGAT2i revealed seven AA differences sites (D3V, A9V, A26P, T37M, T107M, K251R, L316P, Fig. 2), four of which (D3V, A9V, A26P, T37M) were identified with no effect on their enzyme activities [30], and the other three AAs (T107M, K251R, L316P) were thought to determine the enzyme activity. By examine the transmembrane structure we found two potential transmembrane-spanning helices located at the N-terminus (AAs 40–62 and 67–82), interrupted by a very small ER lumen-targeted turn region, and with a large cytosolic C-terminal domain toward cytoplasm [20] (Fig. S1).

### 3.2 Amino acids conservation analysis among DGAT2s from different plant species

Eighty-five DGAT2 proteins from 58 plant species (Table S2) were obtained by blast in NCBI Nr database, and aligned with ClustalW (Fig. S2). Totally 53 AA residues are highly conservative and mainly locate at the middle and C-terminus where the conserved LPLAT_MGAT-like domain locates (AAs 104–321). The three mutant sites (T107M, K251R, L316P) are all located in cytosolic C-terminal domain (Fig. S2). The 107th AA is not conservative, six different AA (Thr, Val, Ile, His, Asn, Cys) are found at this site, in AhDGAT2i it is Met. The 251th AA is not conservative, four different AA (Glu, Lys, Asn, Gln) are found at this site, in AhDGAT2i it is Arg. The 316th AA is highly conservative, it is the same (Leu) in all 85 plant DGAT2s, but in AhDGAT2i it is Pro. So it is speculated that L316P may be the key AA deciding the enzyme activity.

### 3.3 Construction and verification of the plasmids

**Fig. 3:**
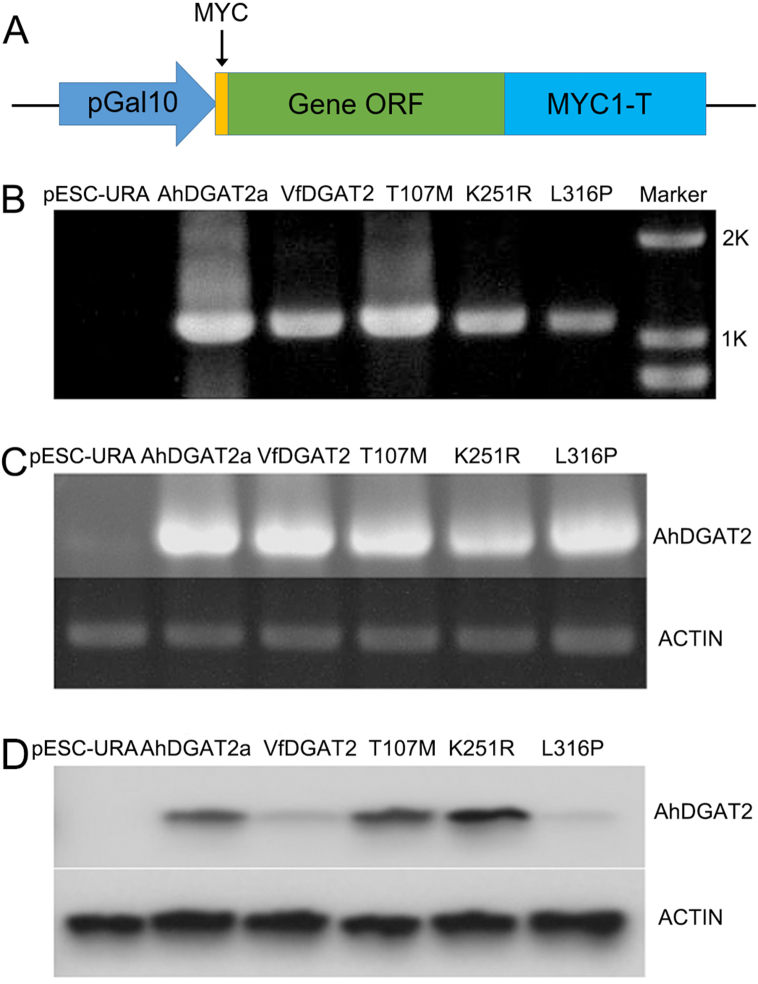
Detection of the transgenic yeast strains. A: Schematic diagram of plasmid structure. B: PCR of the transgenic yeast strains, M: marker. C: RT-PCR of the transgenic yeast strains. D: Western blot of the transgenic yeast strains.

In order to test which one of the three AAs is the decider of the enzyme activity, site-specific mutant sequences were constructed and named as T107M, K251R and L316P, respectively. Each of them has a unique AA difference with AhDGAT2a. T107M, K251R and L316P were cloned into yeast expression vector pESC-URA for function identification, and a myc tag was labeled in N-terminus for western blot analysis (Fig. 3A). AhDGAT2a- and VfDGAT2-transgenic-yeasts were used as the positive controls and pESC-URA-transgenic-yeast as the negative control. The plasmids were successfully constructed. RT-PCR and western blot assay verified that the five transcripts could be expressed at both the transcriptional level (Fig. 3B and C) and the translational level (Fig. 3D). The transgenic yeast strains were used to do the following experiment.

### 3.4 Biochemical activity of the transformed yeast strains

**Fig. 4:**
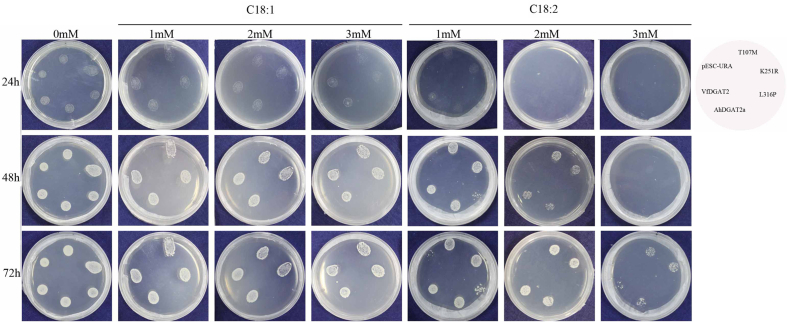
The observations of AhDGAT2a-, T107M-, K251R- and L316P-tansgenic-yeast-strain grown on solid medium supplemented with 1–3 mM oleic or linoleic acid or without FAs at different time period. The empty ESC-URA and VfDGAT2 plasmid were used as the negative and positive control, respectively.

Six transformed yeast strains were cultured on solid medium that contained 1–3 mM oleic or linoleic acid and observed at different time points. An empty pESC-URA plasmid was used as the negative control, and a plasmid that contained VfDGAT2 was used as the positive control. The results showed that the T107M- and K251R-transformed-yeast-strain are highly active, equal to that of AhDGAT2a (Fig. 4). Whereas the L316P-transgenic-yeast-strain could not grow, just as the empty pESC-URA-plasmid-transgenic-yeast-strain.

### 3.5 Nile red assay of the transformed yeast strains

**Fig. 5:**
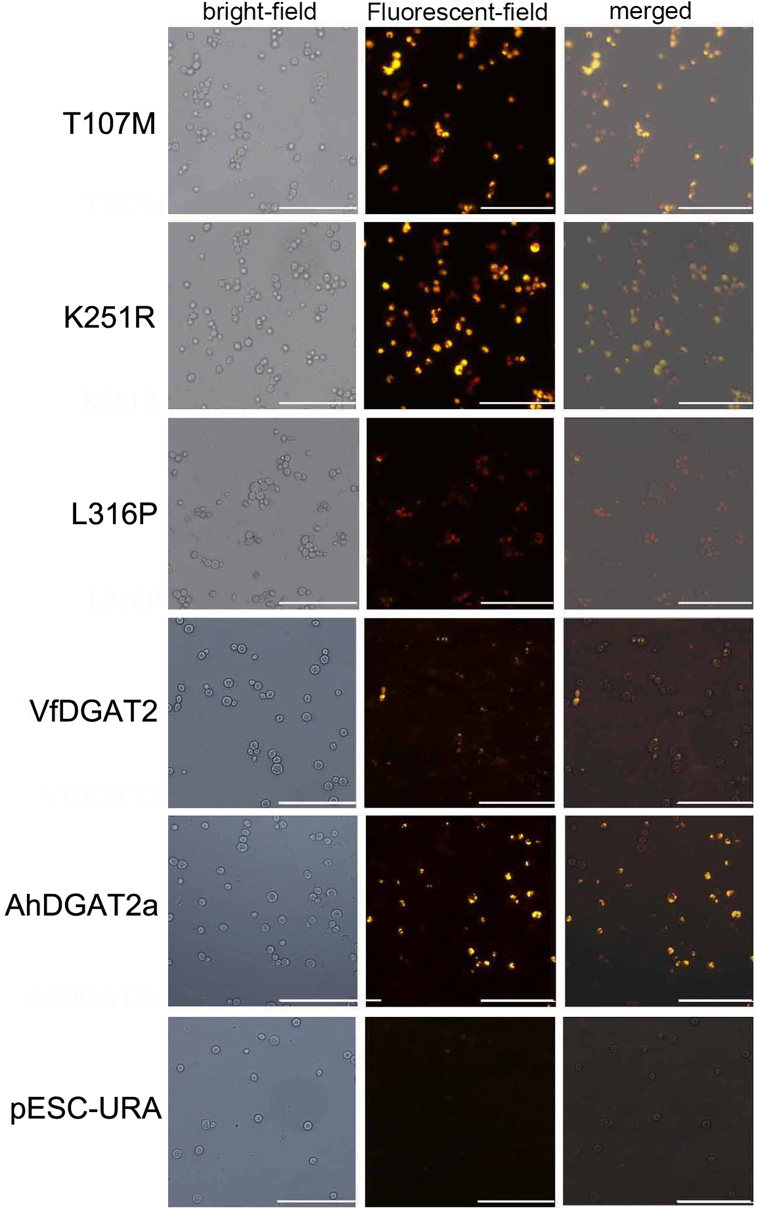
Nile red assay of AhDGAT2a and the mutant tansgenic yeast strains. Bar: 50 μm. (For interpretation of the references to colour in this figure legend, the reader is referred to the Web version of this article.)

The six strains were further analyzed via Nile red staining. The results showed that the T107M-, K251R-, and AhDGAT2a-transgenic-yeast-strain produced large numbers of lipid droplets (Fig. 5). Whereas the L316P-transgenic-yeast-strain could only produce little lipid drops, as the empty pESC-URA-transgenic-yeast-strain.

### 3.6 TLC analysis

**Fig. 6:**
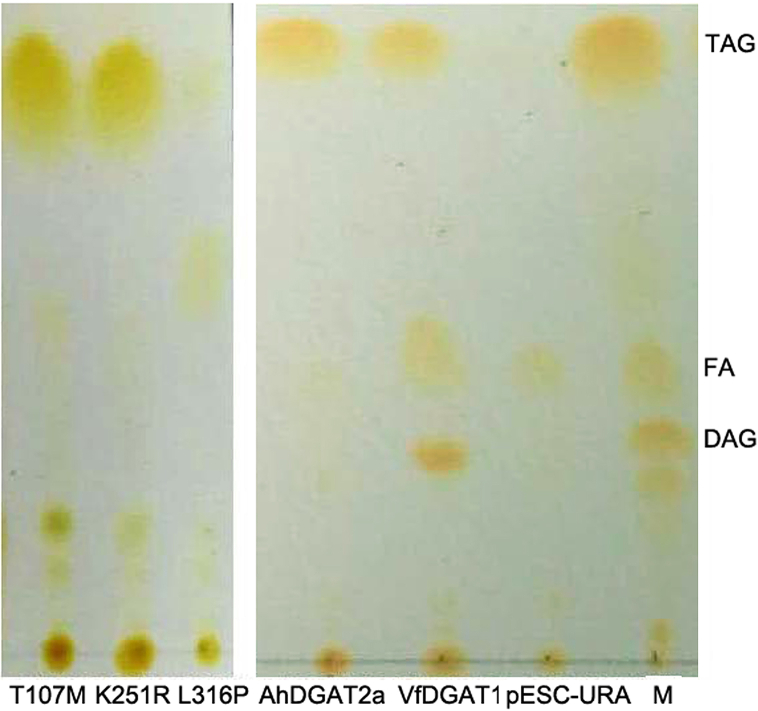
TLC analysis of the six tansgenic yeast strains. TAG, triacylglycerol; FA, free fatty acid; DAG, diacylglyceride; M: marker.

Plant lipid is composed of TAG, diacylglyceride (DAG), and free fatty acid (FFA). A TLC analysis can test the different compositions of lipid. Crude lipids were extracted from the five transgenic yeast strains respectively. TLC results showed that the empty vector and L316P-transgenic-yeast-strain had minimal amounts of TAG, whereas the other strains had large amounts of TAG (Fig. 6).

### 3.7 Comparison of FA composition between transgenic yeast strains

**Fig. 7:**
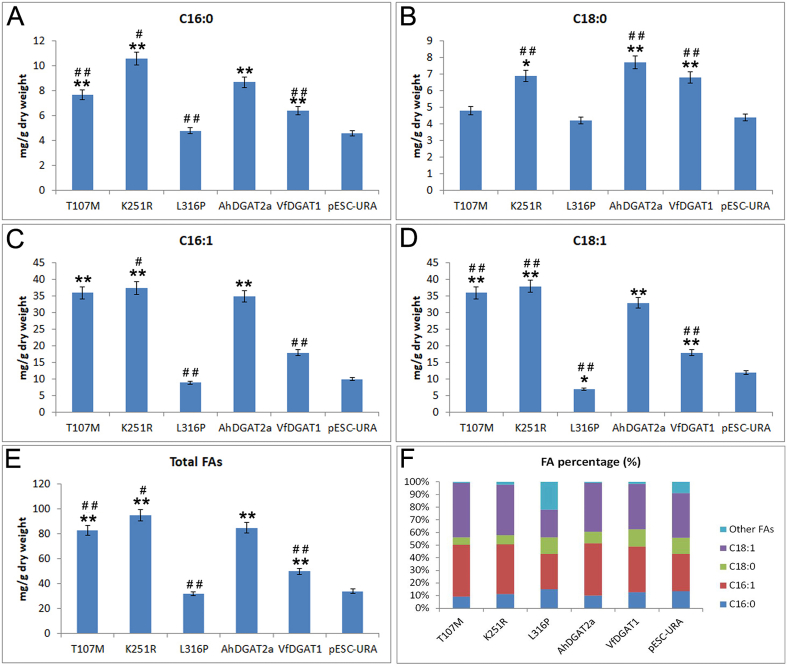
FA content and composition of AhDGAT2-tansgenic-yeast-strains. *: compared with pESC-URA tansgenic yeast strains, *: P < 0.05, **: P < 0.01, #: compared with AhDGAT2a tansgenic yeast strains, #: P < 0.05, ##: P < 0.01. The results were expressed as the mean ± standard deviation (n = 3).

GC was used to examine the FA components of the transgenic yeast strains. Four major FAs were found in yeast lipid, namely, palmitic acid (C16:0), palmitoleic acid (C16:1), stearic acid (C18:0), and oleic acid (C18:1) (Fig. 7).

The results showed that the empty vector and L316P-transgenic-strain has lower FA content than the other transgenic strains. The four major FA amount and total FA amount of L316P-transgenic-yeast-strain are all decreased significantly (Fig. 7A–E), just like the empty-vector-transgenic-strain. The K251R-transgenic-yeast-strain has higher C16:0, C16:1, C18:0 and 18:1 content compared with AhDGAT2a-transgenic-strain (Fig. 7A–D), and leading to the total FA content increased (Fig. 7E). T107M-transgenic-yeast-strain has lower C16:0 and C18:0 amount, but with higher C18:1 amount (Fig. 7A–D), and result in a decrease in total FA content (Fig. 7E). Simultaneously, the FA compositions are different among these transgenic strains (Fig. 7F). AhDAGT2a-, T107M-, K251R- and VfDGAT2-transgenic-strains have similar FA composition, whereas L316P- and pECS-URA-transgenic-strain have similar FA composition. L316P-transgenic-strain has higher C16:0 and C18:0% and lower C16:1 and C18:1%. That is to say, L316P-transgenic-strain has the higher ratio of saturated FAs with the lower ratio of unsaturated FAs.

### 3.8 Amino acid mutations affect the 2D and 3D structure of AhDGAT2a

The crystal structure of DGAT2 has not yet been reported to date and its 2D structure was speculated based on PHYRE2 protein structure prediction software. The results showed that single amino acid mutations lead to the changes in the 2D structure of the proteins, including α-helix and β-folding (Fig. S3). Compared with AhDGAT2a, T107M and K251R has two more β-folds, respectively, and L316P has an extra β-fold.

**Fig. 8:**
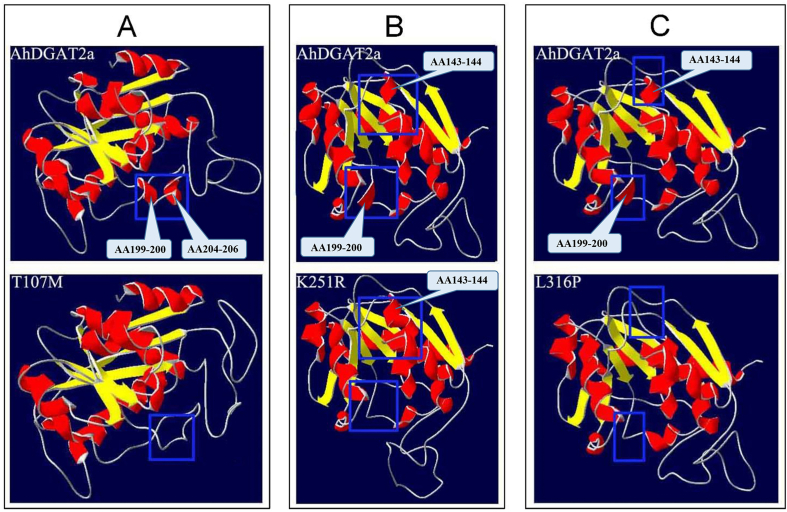
Comparison of the predicted 3D structures between AhDGAT2a and the three mutants. A, AhDGAT2a and T107M; B, AhDGAT2a and K251R; C, AhDGAT2a and L316P.

In order to better understand the influence of the three mutation sites on protein structure, we used swiss-Model online software to predict the 3D structure of the proteins, and further analyzed by swiss-PDB Viewer software. The three mutation sites resulting in the 3D structural differences are the α-helixs of AA143-144, AA199-200 and AA204-206 (Fig. 8). In comparison with AhDGATa, two α-helixs of AA199-200 and AA204-206 disappeared in T107M (Fig. 8A), with no significantly changing the enzyme activity (Figs. 4–7); There are two changes in K251R, a longer α-helix at AA143-144 and the disappearance of α-helix at AA199-200 (Fig. 8B), resulting in increased enzyme activity; The α-helix at AA143-144 and AA199-200 in L316P disappeared (Fig. 8C), resulting in the loss of enzyme activity. Comparing K251R with L316P, the difference lied in the α-helix at AA143-144; in K251R, this α-helix becomes longer and with the increase of the enzyme activity; while in L316P, this α-helix disappeared and with the loss of the enzyme activity. Therefore, it was speculated that the mutant in L316P changed the 3D structure of the enzyme leading to the enzyme inactivation.

## 4 Discussion

Both of DGAT1 and DGAT2 play important role in plant seed lipid synthesis, especially DGAT2. Zhou et al. reported that AtDGAT2 activity showed a double increase in TAG accumulation compared with AtDGAT1 [41]. Peanut genome harbors four classes of DGATs gene families (AhDGAT1, AhDGAT2, AhDGAT3 and WSD/DGAT), and *AhDGAT1* and *AhDGAT2* gene families have four and ten numbers respectively [42]. It seems that AhDGAT2 has a more important function in peanut seed oil accumulation, since AhDGAT2 has a higher expression level compared with AhDGAT1 [42]. But when over-expressed these two genes m in yeast and tobacco, both of them could improve the total FA content of the transformed yeast cells and tobacco seeds at the similar level [30,39], which indicated that both AhDGAT1 and AhDGAT2 have great impact on lipid synthesis in peanut seeds. Furthermore, both of them had no obvious difference in substrate-specificity on oleic and linoleic acid (the two most abundant FAs in peanut oil) [30,39], which was different from other plant DGAT2s, e.g. AtDGAT2 displayed different acyl-CoA substrate preferences than AtDGAT1, AtDGAT2 preferred C16:0, C18:2 and C18:3 [41].

DGAT1 and DGAT2 contain nine and two transmembrane domains (TMDs), respectively, preventing the study on their structure. Till now, the crystal structure and three-dimensional structure of DGAT has not yet been reported and only been speculated based on some protein structure prediction software. Using conservation analysis and site-directed mutagenesis to study the structure–function relationship of DGATs is an effective method. Studies on site-specific mutagenesis of DGAT1 were more than that of DGAT2. Sun et al. identified that five AAs were essential for DGAT1 function and seven other AAs could significantly affect *Chlorella ellipsoidea* DGAT1 function in different degrees [34]. Directed evolution of *B. napus* DGAT1 (BnaDGAT1) revealed that many AA residues are associated with BnaDGAT1 activity, and 67% of these residues are conserved among plant DGAT1s [33]. Further analysis revealed that two variants (L441P and I447F) with AA residue substitutions in PTMD9 result in 33.2 or 70.5% higher TAG content, respectively, relative to native BnaDGAT1 [33]. In maize, a Phe insertion at position 469 of DGAT1 affected the concentrations of seed oil and oleic acid significantly [29].

As to the enzyme active site of DGAT2, the reports was rare. Mishra et al. reported that the mechanism of action of DGAT Type 2B in *Moretierella ramanniana* var. *angulispora* involves a catalytic triad composed of conserved Cys112, His238 and Asn276 [43]. By comparison of two DGAT2 gene homologues of an oleaginous yeast, five AA variations are related to the 2D and 3D structure of CtDGAT2a and CtDGAT2b leading to the different enzyme activity [22]. In this study, we identified three AAs were related to the enzyme activity of AhDGAT2, T107M, K251R and L316P. T107M and K251R resulted in the TAG content increasing while L316P resulted in the TAG content decreased (Figs. 4–7). In L316P, a nonconservative substitution of Leu by Pro resulted in a larger side chain influencing the 2D and 3D structure of the enzyme, thus might block substrate combination and enzyme inactivation.

## 5 Conclusion

Three AAs (T107M, K251R and L316P) were identified to related to the AhDGAT2 enzyme activity, thereinto L316P is the most important, its change will result in the loss of enzyme activity. By analyzing the 2D and 3D structures of the three variants, we found that it is the spatial structure changes that caused the variation of the enzyme activity.

## Declarations

### Author contribution statement

Zhenying Peng: Conceived and designed the experiments; Contributed reagents, materials, analysis tools or data; Wrote the paper.

Ling Zheng, Haiying Tian: Conceived and designed the experiments.

Jianguo Wang, Wenwen Liu, Jingjing Meng: Performed the experiments.

Jialei Zhang: Analyzed and interpreted the data.

Xinguo Li, Shubo Wan: Analyzed and interpreted the data; Contributed reagents, materials, analysis tools or data; Wrote the paper.

### Funding statement

Zhengying Peng was supported by Shandong Provincial Key Research and Development Program [2021LZGC025], Shandong Province Natural Science Foundation project [ZR2020MC057].

Research associate Xinguo Li was supported by National Key R&D Program of China [2018YFD1000900].

### Data availability statement

Data will be made available on request.

### Declaration of interest’s statement

The authors declare no competing interests.

## Appendix A Supplementary data

Multimedia component 1 Primers used in this study. Multimedia component 1

Multimedia component 2 DGAT2s from 58 plant species. Multimedia component 2

figs1 The membrane topology of AhDGAT2i was predicted by TMHMM software with default parameters (http://www.cbs.dtu.dk/services/TMHMM/). The three single site mutations in AhDGAT2i are represented by green dots. figs1

figs2 The sequence alignment of 85 DGAT2 from 58 plant species. figs2

figs3 Secondary structure analysis of three mutant sequences. Red oval: the changed β-fold compared with AhDGAT2a. figs3
