# Supplementary material for: Newly identified essential amino acids affecting peanut (Arachis hypogaea L.) DGAT2 enzyme activity
Source: Heliyon. 2023 Jan 17;9(1):e12878. doi: 10.1016/j.heliyon.2023.e12878 (PMC9876841; doi:10.1016/j.heliyon.2023.e12878)
Supplement: Multimedia component 1 — Primers used in this study. [file mmc1.docx]

Table S1. Primers sequences

| name | sequence |
| --- | --- |
| AhD2FH | 5’ GAATTCAACATGGAAGATCGAGGGAACG 3’ |
| AhD2RH | 5’ CCTTAATTAATCATCAGACAATTCTCAACT 3’ |
| DGAT2T107MR | 5’ TTCAACATGAAGCATGATAGGAAAATAACTG 3’ |
| DGAT2T107MF | 5’ CAGTTATTTTCCTATCATGCTTCATGTTGAA 3’ |
| DGAT2K251RR | 5’ CAAAATTCAGAATTAAACGCCCACCAGGTTTC 3’ |
| DGAT2K251RF | 5’ GAAACCTGGTGGGCGTTTAATTCTGAATTTTG 3’ |
| DGAT2L316PR | 5’TCATCAGACAATTCTCAACTCAAGGTTTGGATATCCAGC  ACGAGCTTTGTACCGTTCAAAAGGATC 3’ |
| yeast actin gene (Genbank No. 850504) | |
| ACTIN-F | 5’ TTCTGGTATGTGTAAAGCCGGTTTT 3’ |
| ACTIN-R | 5’ CAATTTCTCTTTCAGCAGTGGTGGA 3’ |
