# Supplementary figures and images for: Newly identified essential amino acids affecting peanut (Arachis hypogaea L.) DGAT2 enzyme activity
Source: Heliyon. 2023 Jan 17;9(1):e12878. doi: 10.1016/j.heliyon.2023.e12878 (PMC9876841; doi:10.1016/j.heliyon.2023.e12878)

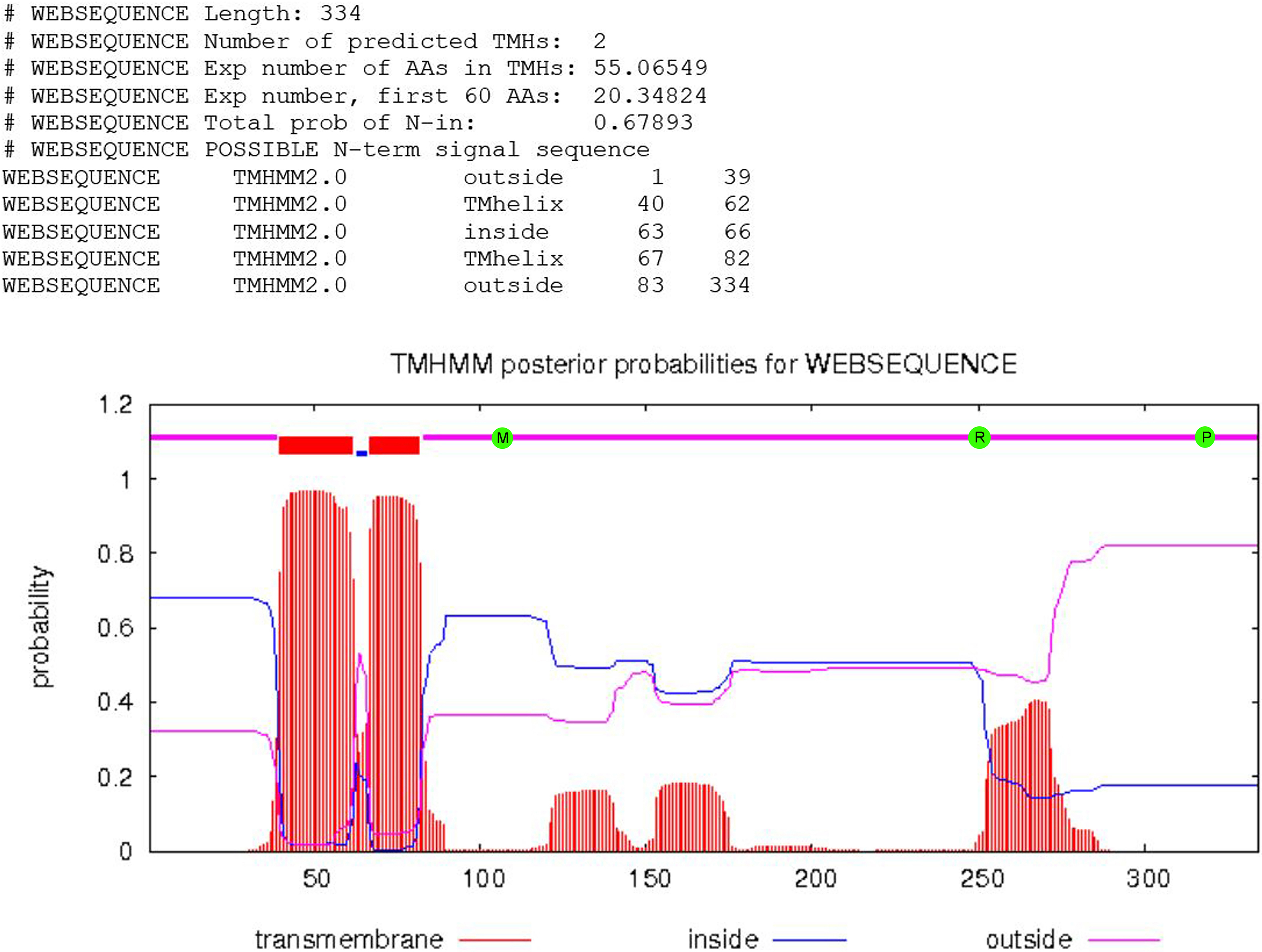

Supplement: figs1 — The membrane topology of AhDGAT2i was predicted by TMHMM software with default parameters (http://www.cbs.dtu.dk/services/TMHMM/). The three single site mutations in AhDGAT2i are represented by green dots. [file mmcfigs1.jpg]

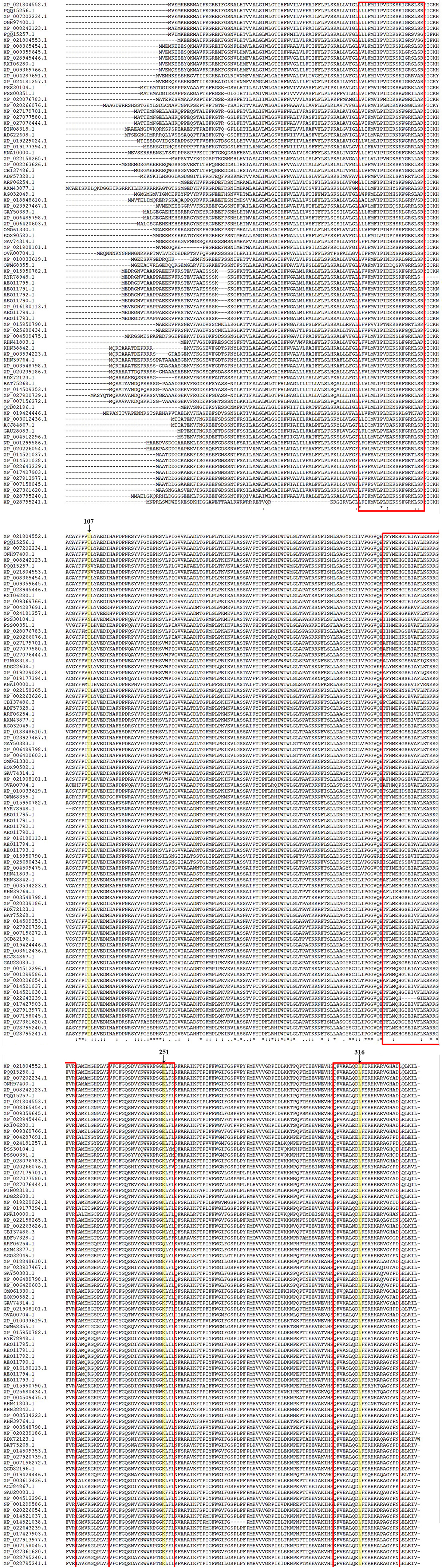

Supplement: figs2 — The sequence alignment of 85 DGAT2 from 58 plant species. [file mmcfigs2.jpg]

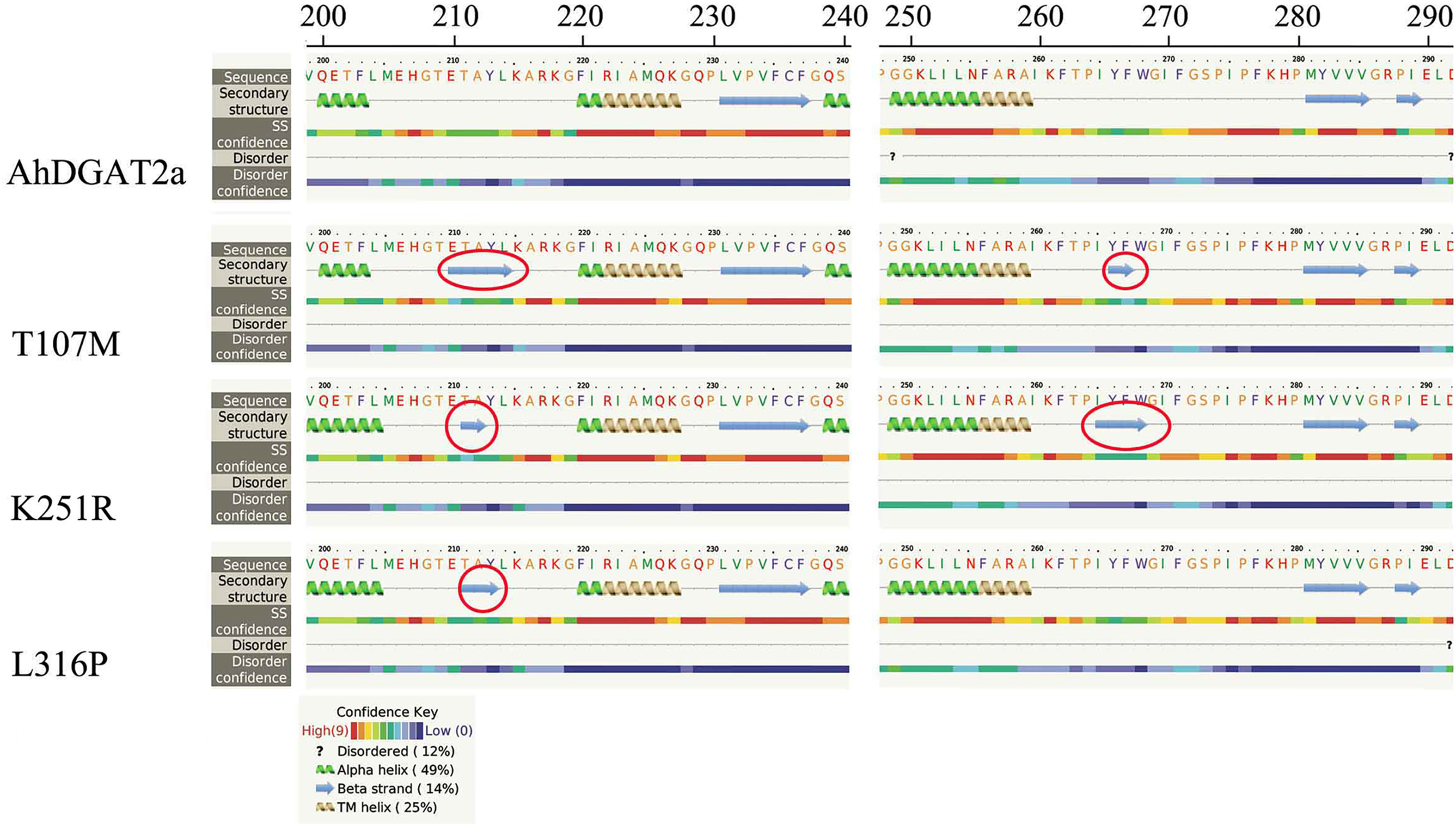

Supplement: figs3 — Secondary structure analysis of three mutant sequences. Red oval: the changed β-fold compared with AhDGAT2a. [file mmcfigs3.jpg]
